# Familial hemophagocytic lymphohistiocytosis in a neonate: Case report and literature review

**DOI:** 10.1097/MD.0000000000027786

**Published:** 2021-11-24

**Authors:** Yue Yang, Zebin Luo, Tianming Yuan

**Affiliations:** a Department of Neonatology, Children's Hospital, Zhejiang University School of Medicine, National Clinical Research Center for Child Health, Zhejiang, China; b Department of Hematology, Children's Hospital, Zhejiang University School of Medicine, National Clinical Research Center for Child Health, Zhejiang, China.

**Keywords:** case report, cytokines, familial hemophagocytic lymphohistiocytosis, hemophagocytic lymphohistiocytosis, neonatal thrombocytopenia

## Abstract

**Rationale:**

Familial hemophagocytic lymphohistiocytosis (FHL) is a potentially fatal disease that rarely presents in the neonatal period. Timely diagnosis is a key challenge owing to the atypical clinical manifestations. Here, we describe a case of FHL type 3 with disease onset in the early neonatal period and review the relevant literature. Our findings may provide insights into the diagnosis and treatment of this rare disease.

**Patient concerns:**

A 6-day-old male neonate presented with fever, hepatosplenomegaly, cytopenia, hyperferritinemia, hypofibrinogenemia, hemophagocytosis, and hypertriglyceridemia.

**Diagnosis:**

Considering the clinical picture (prolonged fever, progressive hepatosplenomegaly, high triglycerides, low fibrinogen, and high ferritin), along with abnormal natural killer-cell activity, combining sequence analysis of genomic DNA results (compound heterozygous mutations of *UNC13D*), the patient was finally diagnosed with FHL type 3 (FHL3).

**Interventions:**

The patient was initially treated with HLH-1994 protocol and subsequently switched to an oral regimen of ruxolitinib due to incomplete remission of the disease.

**Outcomes:**

The trend of change in weekly cytokine levels, neutrophil counts, hemoglobin, and platelet counts indicated that the complete remission was not achieved after the treatment of HLH-1994 protocol. The platelet counts fluctuated within the normal range after oral administration of ruxolitinib. But soon after, the patient did not respond to treatment and eventually died of respiratory failure.

**Lesson:**

Timely diagnosis of FHL is challenging. This case report illustrates that thrombocytopenia can be the first clinical sign of FHL with neonatal onset. Genetic testing, detection of cytokines, and flow cytometry should be performed as soon as possible to confirm the diagnosis. Given the high morbidity and mortality of FHL, pediatricians should have a high suspicion index for this disease.

## 1 Introduction

Hemophagocytic lymphohistiocytosis (HLH) is a potentially fatal disease characterized by dysregulated immune response to antigens, resulting in uncontrolled activation of immune cells and life-threatening cytokine storm.^[1,2]^ HLH presents in a variety of clinical contexts and with multiple etiologic associations. HLH can be categorized into 2 types: primary and secondary. The term “primary HLH” often refers to patients with clear familial inheritance or genetic causes. Patients in the “secondary HLH” category are those associated with infections, autoimmune disease, or underlying malignancy.^[3]^ The main clinical manifestations are fever, hepatosplenomegaly, and cytopenia, while some patients may exhibit central nervous system involvement.^[2]^ Although HLH can occur in all age groups, neonatal onset is rare, accounting for only 4% of all HLH cases.^[4]^ Autosomal recessive inheritance is a common mode of inheritance of this disease [also known as familial hemophagocytic lymphohistiocytosis (FHL)]. Most children with FHL are born healthy and develop the illness in the first 2 to 6 months of life.^[5]^ FHL with onset in the neonatal period is rare. Here, we describe a case of FHL type 3 with disease onset in the early neonatal period and review the relevant literature. Our findings may provide insights into the diagnosis and treatment of this rare disease.

## 2 Case presentation

A 6-day-old male neonate was referred to the neonatal department of our hospital on November 30, 2019 because of moaning and rash since the last 5 days. He was delivered by elective caesarean section at a local hospital (gestational age: 40 weeks; birth weight: 4100 g). Apgar scores at 1 and 5 minutes were both 10. His parents denied close consanguineous marriage. The patient started moaning and exhibited shortness of breath after birth, with pinkish unfading rash after pressing on face; there was no history of fever.

At the local hospital, blood routine (day of life [DOL] 1) examination showed normal white blood cell (WBC) count (7.2 × 10^9^/L), neutrophil count (4.12 × 10^9^/L), and hemoglobin level (140 g/L); however, he had severe thrombocytopenia (27 × 10^9^/L). Chest x-ray (DOL1) showed inflammatory changes in both lower lung fields. With a presumptive diagnosis of neonatal pneumonia and thrombocytopenia, he was treated with penicillin, cephalosporin, vitamin K1, dexamethasone, intravenous immunoglobulin, platelet transfusion, and oxygen inhalation. After treatment, his respiratory symptoms were alleviated; however, the platelet count was still in the range of 10 to 47 × 10^9^/L.

**Figure 1:**
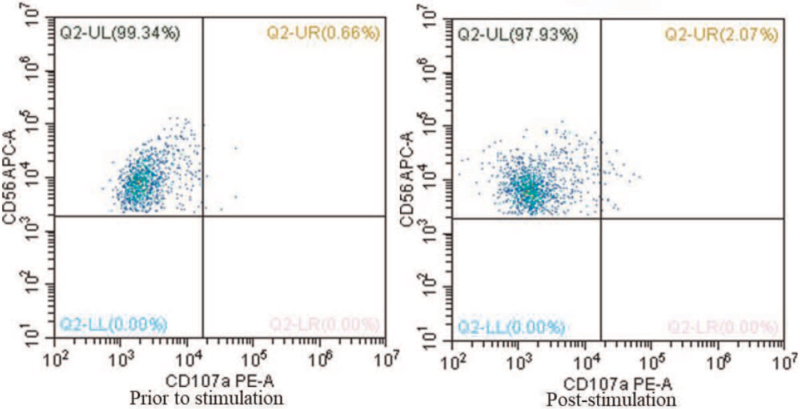
Severely decreased ΔCD107a in NK cells of the patient. The expression of CD107a in NK cells is 0.66% prior to stimulation and 2.07% poststimulation; thus, ΔCD107a is 1.41% (ΔCD107a >10% indicates normal degranulation function). NK = natural killer.

**Figure 2:**
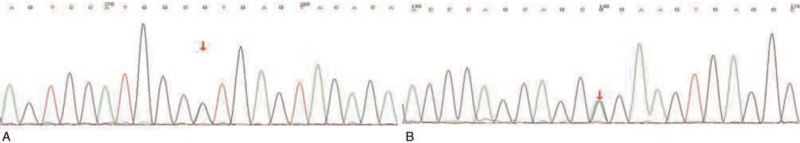
Compound heterozygous pathogenic mutation of *UNC13D* in the patient: c. 1055+1G>A in exon12 (A) and c. 118-308C>T in intron (B) have been identified.

**Figure 3:**
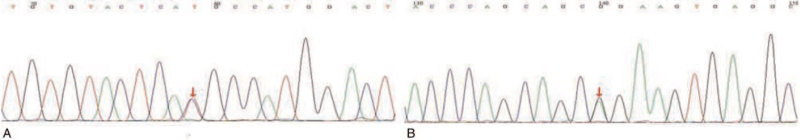
Sanger sequencing confirmed that c. 1055+1G>A in exon12 was inherited from the patient's father (A) and c. 118-308C>T in intron was inherited from his mother (B).

Subsequently, he was admitted to our neonatal department on DOL6 for further management due to persistent thrombocytopenia. On physical examination, his body temperature was 37.5°C; liver was palpable at 2 cm below the rib edge, while spleen was not palpable; pressing on the face resulted in a pinkish unfading rash. On DOL9, he developed supraventricular tachycardia, which responded to treatment with propafenone. Blood routine examination (1 hour after admission at our hospital) showed decreased WBC (5.53 × 10^9^/L) and neutrophil counts (0.78 × 10^9^/L), reduced hemoglobin (110 g/L), and low platelet count (36 × 10^9^/L). Routine urine and fecal examination were normal. Tests for Epstein-Barr virus, respiratory pathogens, enterovirus, and TORCH test (including toxoplasmosis, syphilis, hepatitis B, rubella, cytomegalovirus, and herpes simplex virus) were negative. Sputum culture and blood culture were also negative. The antinuclear antibody test showed positivity only for anti-Ro/SS-A. His mother's antinuclear antibody was negative and she had no signs of any autoimmune disease. Magnetic resonance imaging of skull revealed no obvious abnormality. Bone marrow biopsy showed actively proliferating nucleated cells and there were no abnormal cells. At that time, a provisional diagnosis of “neonatal thrombocytopenia, hemocytopenia, and neonatal septicemia” was considered. After supportive treatment, platelet count increased to 70 × 10^9^/L (DOL13). On DOL14, he developed persistent high fever (37.8–39°C) with progressive enlargement of liver and spleen (on ultra-sound examination, liver was 4 cm below the rib edge and spleen was 3.8 cm below the rib edge), cytopenia (WBC: 3.57 × 10^9^/L, neutrophils: 0.17 × 10^9^/L, hemoglobin: 69 g/L, platelet count: 8 × 10^9^/L), increased ferritin (>1500 mg/L), decreased fibrinogen (0.95 g/L), high triglycerides (4.81 mmol/L), and elevated cytokines (interferon [IFN]-γ: 73.6 pg/mL; interleukin [IL]-10: 1081.9 pg/mL; and IL-6: 100.1 pg/mL). Considering the clinical picture (prolonged fever, progressive hepatosplenomegaly, high triglycerides, low fibrinogen, and high ferritin), which qualified 5 out of the 8 HLH-2004 criteria,^[6]^ the diagnosis of HLH was confirmed and the patient was transferred to the hematology department for supportive treatment with HLH-1994 protocol. Subsequent natural killer (NK) cells stimulation test showed severe dysregulation of immune response (Fig. 1). Sequence analysis of genomic DNA from his blood sample revealed compound heterozygous mutations of *UNC13D* gene: c.1055+1G>A in exon12 (pathogenetic mutation) and c.118-308C>T in intron (likely pathogenetic mutation). Genetic testing of parents confirmed that the mutation c.1055+1G>A in exon12 was inherited from his father and c.118-308C>T in intron from his mother (Figs. 2 and 3). Based on the above results, the patient was finally diagnosed with FHL type 3 (FHL3).

The patient was discharged on January 5, 2020 and followed up in the hematology department with weekly etoposide (VP-16) infusion and adjustment of dexamethasone dose according to the HLH-1994 protocol. The trend of change in cytokine levels, neutrophil counts, hemoglobin, and platelet counts since the onset of HLH are shown in Table 1. Laboratory examination after 8 weeks of standard chemotherapy showed normal WBC count (8.15 × 10^9^/L), neutrophil count (1.85 × 10^9^), thrombocytopenia (67 × 10^9^/L), low fibrinogen (1.55 g/L), high triglycerides (5.06 mmol/L), and normal cytokine levels (IFN-γ: 3.3 pg/mL, IL-10: 43.3 pg/mL, IL-6: 12.2 pg/mL). Spleen was still 3.6 cm below the ribs. These findings indicated that complete remission was not achieved. Subsequently, he was prescribed oral ruxolitinib. The platelet counts fluctuated within the normal range for some time. However, the patient presented again with fever (37.9°C) and hypercytokinemia (IFN-γ: 58.6 pg/mL; IL-10: 260.3 pg/mL; IL-6: 17.5 pg/mL) on March 30, 2020. The patient did not respond to treatment and eventually died of respiratory failure.

**Table 1:** Trend of change in cytokine levels (IL-10, IFN-γ, IL-6) (pg/mL), neutrophil count (×10^9^/L), hemoglobin level (g/L), and platelet count (×10^9^/L) since the onset of hemophagocytic lymphohistiocytosis.

Day of life (DOL)	Neutrophil (1.5–7.8)	Hemoglobin (110–155)	Platelet (100–400)	IL-10 (2.6–4.9)	IFN-γ (1.6–13.7)	IL-6 (1.7–16.6)
6	0.78	110	36			
19	0.17	100	8	1081.9	73.6	100.1
46	2.93	96	560	51.1	5.1	4.4
84	1.85	88	67	43	3.3	12.2
98	2.41	96	425			
113	0.25	81	113	260.3	58.6	17.5
119	0.96	97	105	79.8	34.8	6.3
129	1.23	55	193	365.8	110.3	4.9
136	0.57	81	71	82.6	510	22.6
143	2.54	49	117	562.5	44.3	17.6
147	4.48	100	8			
155	1.74	65	60	10.8	12.8	7.5
159	2.4	51	5			
163	0.89	117	13	25.6	24.5	9.3
171	9.82	92	11			

IL-10 = interleukin-10, IFN-γ = interferon-γ, IL-6 = interleukin-6.

## 3 Literature review and discussion

HLH is a potentially life-threatening pathological immune activation syndrome characterized by functional defects of cytotoxic T lymphocytes and NK cells. HLH patients develop systemic inflammation due to uncontrolled production of inflammatory cytokines and macrophage activation.^[7]^ HLH is categorized into 2 types: primary or secondary. It can be inherited or may occur secondary to infections, autoimmune disease, or underlying malignancy. There is a lack of nationwide data in China, and the literature reports mainly pertain to secondary HLH. In a multicenter retrospective study by Xu et al,^[2]^ the median age of children (n = 323) at diagnosis of HLH was 2.2 years. With an incidence of approximately 1:50,000 to 1:300,000 live births, most children with FHL are born healthy and develop symptoms in the first 2 to 6 months of life, although it can occur in all age-groups, neonatal onset is rare, accounting for only 4% of all HLH cases.^[8,9]^

Here, we reviewed 9 cases of neonate FHL (onset within 28 days after birth) (Table 2).^[10–18]^ This table shows the clinical and laboratory features of 9 infants. These cases were all identified as FHL because of positive family history or the discovery of related gene mutations. Four cases were FHL type2, 2 cases were FHL type5, only 1 case was FHL type3, and the other 2 cases did not find the specific gene mutations. As we can see, neonatal HLH has an atypical presentation, fever, hepatosplenomegaly, and thrombocytopenia were almost consistently found in all cases. In some cases, non-specific initial clinical symptoms such as non-immune hydrops and fetal distress are the only manifestation. Therefore, timely diagnosis of neonatal HLH is typically challenging, especially in infants with fever and thrombocytopenia. Neonatal thrombocytopenia is a common problem in all infants. As the diagnostic criteria for HLH include cytopenia, HLH should be considered in infants with thrombocytopenia, especially in those presenting with multiple organ involvement or elevated cytokine levels.^[15]^ In a retrospective analysis of 124 children with FHL, thrombocytopenia was observed in all patients.^[19]^ Fever of unknown reason is another common clinical manifestation of HLH, which needs to be differentiated from that caused by secondary infection. Clinically, extremely elevated IL-6 (>1000 pg/mL) and slightly elevated IFN-γ are mostly seen in sepsis caused by gram-negative bacteria. On the contrast, Xu et al^[20]^ found that in the acute phase of HLH, the levels of IFN-γ and IL-10 were significantly increased, while those of IL-6 were only moderately increased, the same cytokine changes were found in the fourth case in Table 2. Cytokine profile can be an important biomarker for monitoring HLH status, which can allow for earlier diagnosis and institution of more effective treatment.^[13]^ In our patient, the level of IFN-γ and IL-10 gradually decreased to normal range after treatment with the HLH-1994 protocol and oral ruxolitinib, which indicated that the disease was in remission. However, the patient presented again with fever, pulmonary pneumocystis, elevated IFN-γ and IL-10, which resulted from the re-activation of HLH (Table 1).

**Table 2:** List of cases related to neonatal familial hemophagocytic lymphohistiocytosis.

No.	Study	Age of onset	Symptoms and signs	Laboratory examination	Gene mutation	Treatment	Outcomes
1	Roganović et al (2010)^[10]^	1DOL	Respiratory distress, jaundice, fever, hepatosplenomegaly, subcutaneous nodules	Thrombocytopenia, anemia, high ferritin, hypofibrinogenemia	Not found	HLH-04 protocol	Died
2	Jain et al (2012)^[11]^	21DOL	Fever, hepatosplenomegaly, abdominal distension, lethargy	Thrombocytopenia, anemia, high ferritin, hypofibrinogenemia, hypertriglyceridemia	c.1697G>A mutation in STXBP2 gene	Dexa, CsA	Died
3	Chia et al (2012)^[12]^	1DOL	Fever, hepatosplenomegaly, ascites	Cytopenia, high ferritin, elevated liver enzymes, hypofibrinogenemia	c.145G>A in exon2 and c.[853_855delAAG] in exon3 in PRF1 gene	HLH- 04 protocol	Died
4	Chen et al (2013)^[13]^	8DOL	Fever, hepatosplenomegaly,	Thrombocytopenia, high ferritin, hypofibrinogenemia, hypertriglyceridemia, low perforin expression, degranulation defect of NK cells, increased IFN-γ and IL-10	c.2295_2298delGCAG, p.Glu765Aspfs∗27 mutations in exon 23 of UNC13D gene	HLH-04 protocol	Died
5	Greenhalgh et al (2015)^[14]^	1DOL	Respiratory distress, abdominal distension, hepatosplenomegaly, jaundice, seizure, hypotension, hypoglycemia	Thrombocytopenia, anemia, high ferritin, elevated liver enzymes	Compound heterozygous-ity mutations in PRF1 gene	Blood transfusion	Died
6	Hinson et al (2015)^[15]^	1DOL	Petechia, hepatosplenomegaly, edema, fever, scleral icterus, silvery hair, generalized edema	Thrombocytopenia, elevated liver enzymes, high ferritin, elevated sIL2r	Allele 1:272C>T (A91V) in PRF1 gene	HLH-04 protocol, blood transplant	Alive
7	Zarrini et al (2017)^[16]^	2DOL	Respiratory distress, fever, hepatosplenomegaly, jaundice	Cytopenia, high ferritin, elevated liver enzymes, hypofibrinogenemia, hypertriglyceridemia,	Not found	HLH-04 protocol	Died
8	Sato et al (2020)^[17]^	18DOL	Fever, hepatosplenomegaly, petechia	Cytopenia, elevated liver enzymes, hypofibrinogenemia	c.781G>A (p.E261K) and c.1491T>A (p.C497∗) in exon 3 of PRF1 gene	Prednisolone	Died
9	Benavides et al (2020)^[18]^	1DOL	hepatosplenomegaly	Hyperbilirubinemia, thrombocytopenia, anemia, high ferritin, elevated sIL2r, elevated liver enzymes, degranulation defect of NK cells	c.568C>T (p.Arg190Cys) in exon 7 of STXBP2 gene	Dexa, VP-16, CsA	Alive

CsA = cyclosporine A, Dexa = dexamethasone, DOL = day-of-life, HLH = hemophagocytic lymphohistiocytosis, IFN-γ = interferon-γ, IL-10 = interleukin-10, NK = natural killer, VP-16 = etoposide.

There are many genetic causes of HLH susceptibility. The cause of FHL type 1 remains unclear. FHL types 2 to 5 are due to mutations in PRF1, UNC13D, STX11, and STXBP2, respectively. PRF1 and UNC13D mutations were the most common mutations in Chinese children with FHL.^[21]^ The protein products of these genes play an important role in normal cytotoxic granule exocytosis. In FHL patients, granules contents (containing perforin and granzymes) will not be released into the immune synapse, and the target cells will not be killed.^[22]^ FHL type3 (FHL3) is caused by a mutation in the *UNC13D* gene encoding Munc13-4 protein, located in 17q25. Till date, at least 112 different mutations in *UNC13D* have been reported as the cause of FHL3: 60 missense/non-sense, 25 splicings/regulatory, 25 deletion/insertion mutations, and 2 complex gene rearrangements.^[23]^ The possibility of a potential genetic diagnosis is highest in infants. In a study of Chinn et al,^[24]^ 61% of patients below the age of 1 year were found to have a genetic HLH disease. Gene testing of our patient showed that the UNC13D gene carried a compound heterozygous mutation, from father and mother (respectively), which has been reported as a pathogenic mutation site in the past.^[25]^

Other than mutation analysis, flow cytometric measurements are also important to further verify the pathogenetic effect of gene mutation. The results of our patient showed that the cytotoxicity of NK cells was normal; and the expression of CD107a was greatly decreased, which was consistent with the flow cytometry results in patients with FHL3.^[26]^ However, due to incomprehensive medical technology and the rapid development of the patient's condition, the detection of NK cells degranulation function failed to start in time in most cases.

As shown in Table 2, 4 patients received HLH-2004 protocol, but only 2 patients survived after treatment, indicating a high mortality rate of neonate HLH. The prognosis for HLH infants is poor if untreated. A commonly used treatment approach consists of dexamethasone and VP-16 based on the experiences of the Histiocyte Society HLH-1994 and HLH-2004 studies.^[22]^ Our patient did not achieve complete remission after 8 weeks of application of the HLH-1994 protocol, which suggested that the disease was still in active stage. Notably, some novel treatment regimens have been reported to further improve the efficiency of treatment. A retrospective study suggested that alemtuzumab therapy of refractory HLH resulted in improvement and survival to allogeneic hematopoietic stem cell transplantation (HSCT) in most patients.^[27]^ In the study of Bami et al,^[28]^ initial treatment with anakinra is a feasible treatment alternative for patients with secondary HLH and may allow for avoidance of VP-16. Wang et al^[29]^ reported a response rate up to 75% in refractory HLH achieved by using the liposomal doxorubicin treatment combined with VP-16 and methylprednisolone as salvage therapy. Emapalumab was an efficacious targeted therapy for patients with primary HLH, Franco et al^[30,31]^ found that across all patients treated with emapalumab (n = 34), the overall response rate at the end of treatment was 64.7%. Our patient was treated with oral ruxolitinib as a second-line treatment, and the platelet counts fluctuated within the range of 300 to 500 × 10^9^/L for some time. Ruxolitinib is a safe and effective salvage therapy that alleviates HLH symptoms by reducing cytokine levels and increases the possibility of refractory/recurrent HLH patients receiving HSCT or treatment for underlying diseases.^[32]^ Consequently, a multicenter, non-randomized trial to investigate the efficacy of ruxolitinib combined with the doxorubicin-etoposide-methylprednisolone regimen as a salvage therapy for refractory/relapsed HLH is underway in China.^[33]^ In most cases of FHL, immune-chemotherapy can temporarily control the disease; however, the disease is eventually fatal unless HSCT is performed. Unfortunately, our patient presented with pneumocystis, which resulted in respiratory failure, cardiac arrest, and other serious complications prior to receiving HSCT.

## 4 Conclusion

We report an infant with FHL3 who presented with thrombocytopenia as the initial clinical symptom. FHL with onset during the neonatal period is associated with high mortality and morbidity. Prompt recognition and treatment are critical to increasing the likelihood of survival. Owing to the non-specific clinical symptoms of neonatal FHL, timely diagnosis and treatment are typically challenging. In most FHL infants, thrombocytopenia is the initial clinical manifestation, while other symptoms such as fever and elevation of cytokines appear as the disease progresses. Neonatal FHL can rapidly deteriorate and lead to death in the absence of timely treatment. Therefore, the possibility of FHL should be considered in newborns who develop worsening thrombocytopenia of unknown etiology. Detection of cytokine levels and NK cells degranulation function should be performed as soon as possible. In cases with high clinical suspicion of neonatal HLH, treatment should be initiated even if the results of genetic testing are awaited.

## Author contributions

**Conceptualization:** Tianming Yuan, Zebin Luo.

**Data curation:** Tianming Yuan, Yue Yang, Zebin Luo.

**Formal analysis:** Yue Yang.

**Funding acquisition:** Tianming Yuan.

**Resources:** Tianming Yuan, Zebin Luo.

**Supervision:** Tianming Yuan.

**Writing – original draft:** Yue Yang, Zebin Luo.

**Writing – review & editing:** Tianming Yuan, Zebin Luo.
